# Microscopic evaluation of microleakage in retrograde filling materials using conventional and ultrasonic techniques

**DOI:** 10.6026/973206300214570

**Published:** 2025-12-15

**Authors:** Ramsha Rizwan, Deepak Kurup, Shazia Mahreen, Shreya Saurabh, Garima Sinha, Sourav Tikadar

**Affiliations:** 1 Department of Conservative Dentistry and Endodontics, Hazaribag College of Dental Sciences and Hospital, Demotand, Hazaribag, Jharkhand, India

**Keywords:** Microleakage, retrograde filling, mineral trioxide aggregate (MTA), Biodentine, One-Fil PT, ultrasonic preparation, conventional bur

## Abstract

Retrograde filling success largely depends on the sealing ability of the material and the quality of the cavity preparation technique. Therefore, it is of interest to compare apical microleakage among MTA, Biodentine and One-Fil PT using conventional bur and ultrasonic retrograde cavity preparation. One hundred prepared teeth were restored, dye-immersed, sectioned and evaluated under a stereomicroscope for leakage. MTA showed the least microleakage, followed by Biodentine, while One-Fil PT exhibited significantly higher leakage. Within study limitations, MTA remains the most effective retrograde filling material, with ultrasonic preparation offering additional sealing benefits.

## Background:

Microleakage, defined as the clinically undetectable passage of bacteria, fluids, molecules, or ions between the tooth structure and restorative material, remains a significant challenge in endodontic therapy [1]. In surgical endodontics, the prevention of microleakage at the root-end filling interface is critical for long-term success, as it can lead to persistent periapical inflammation and treatment failure [2]. The quality of the apical seal depends on both the preparation technique of the root-end cavity and the physical and chemical properties of the retrograde filling material [3]. Apical surgery, first described by Farrar in 1884, has evolved significantly with the introduction of microsurgical techniques and advanced materials [4]. The objective of modern apical surgery is to maintain teeth with endodontic lesions that cannot be resolved by conventional endodontic treatment, while ensuring a fluid-tight seal of the root canal system to promote periapical healing [5]. The ideal retrograde filling material should possess several properties, including biocompatibility, dimensional stability, non-toxicity, radiopacity, non-resorbability and the ability to stimulate tissue repair [6]. Various materials have been used for retrograde fillings over the years, including amalgam, glass ionomer cement, composite resins and more recently, mineral trioxide aggregate (MTA) and other bioactive ceramics [7]. MTA, introduced in the 1990s, has become the gold standard for retrograde filling due to its excellent sealing ability, biocompatibility and regenerative potential [8]. Biodentine, a tricalcium silicate-based material introduced in 2011, has shown promising results as an alternative to MTA, with the advantages of shorter setting time and improved handling properties [9]. One-Fil PT is a more recently introduced tricalcium silicate-based material that requires further investigation regarding its sealing ability [10].

The technique used for root-end cavity preparation also plays a crucial role in the quality of the apical seal. Traditional preparation using conventional burs in slow-speed handpieces often results in irregular cavity walls and significant smear layer formation [11]. In contrast, ultrasonic retrotips produce more precise cavities with less debris and better access, potentially improving the adaptation of the filling material [12]. Several studies have compared these techniques, with most reporting superior results with ultrasonic preparation [13]. Despite numerous studies on retrograde filling materials and preparation techniques, there remains a need for comparative evaluations of newer materials like One-Fil PT and direct comparisons between MTA and Biodentine using different preparation techniques [14]. Furthermore, the interaction between preparation techniques and different filling materials has not been fully elucidated [15]. Therefore, it is of interest to evaluate and compare the apical microleakage of root-end cavities filled with MTA, Biodentine and One-Fil PT, using two different cavity preparation techniques: conventional bur preparation and ultrasonic tip preparation.

## Materials and Methods:

## Study design:

This *in vitro* study was designed to evaluate and compare the microleakage of three different retrograde filling materials using two different cavity preparation techniques. The study protocol was approved by the Institutional Ethics Committee (Reference No: IEC/2021/045)

## Sample selection:

A total of 100 extracted single-rooted human teeth (maxillary central incisors, maxillary lateral incisors and mandibular premolars) were collected for the study. Teeth were included if they had fully developed apices, no visible cracks or fractures, no previous endodontic treatment, no root caries and no resorption defects. Teeth with curved roots (more than 20 degrees), calcified canals, or anomalies were excluded. The collected teeth were cleaned of soft tissue and calculus using hand scalers and stored in 0.1% thymol solution at 4°C until use.

## Sample preparation:

All teeth were decoronated at the cementoenamel junction (CEJ) using a diamond disc under water coolant to standardize the root length to 15mm. Canal patency was established using#15 K-file (Dentsply Maillefer, Ballaigues, Switzerland). Working length was determined by subtracting 1mm from the length at which the file tip was visible at the apical foramen. Biomechanical preparation was performed using the step-back technique up to a master apical file size #50 K-file. Irrigation was performed with 3% sodium hypochlorite (NaOCl) and 17% EDTA alternately during instrumentation. A final rinse with 5ml of 17% EDTA for 1 minute followed by 5ml of 3% NaOCl for 1 minute was performed to remove the smear layer. The canals were then dried with paper points. All samples were stored in an incubator at 37°C and 100% humidity for 5 weeks to simulate the oral environment. Canals were obturated with gutta-percha cones (Dentsply Maillefer, Ballaigues, Switzerland) using the lateral compaction technique and AH Plus sealer (Dentsply DeTrey, Konstanz, Germany). Access cavities were sealed with composite resin (Beautifil II, Shofu, Kyoto, Japan) after 24 hours. The teeth were then incubated for one week at 37°C and 100% humidity.

## Apical resection and retrograde cavity preparation:

The apical 3mm of each root was resected at a 90-degree angle to the long axis of the root using a cross-cut fissure bur (SS White Burs, Lakewood, NJ, USA) under water coolant.

The samples were randomly divided into four experimental groups (n=25) based on the retrograde filling material to be used:

[1] Group I: MTA (Angelus, Londrina, PR, Brazil)

[2] Group II: Biodentine (Septodont, Saint-Maur-des-Fossés, France)

[3] Group III: One-Fil PT (Mediclus, Cheongju, Korea)

[4] Group IV: Unfilled control group

Each group was further divided into two subgroups (n=12) based on the cavity preparation technique:

[1] Subgroup a: Ultrasonic retrotip preparation

[2] Subgroup b: Conventional bur preparation

For conventional bur preparation, a small round bur (#6) (SS White Burs, Lakewood, NJ, USA) was used in a slow-speed handpiece at 10,000 rpm under water coolant. The cavity was prepared to a depth of 3mm parallel to the long axis of the root.

For ultrasonic preparation, an E11D scaler tip (Woodpecker, Guilin, China) was used on a UDS-P ultrasonic scaler unit (Woodpecker, Guilin, China) at medium power setting as recommended by the manufacturer. The cavity was prepared to a depth of 3mm parallel to the long axis of the root. After preparation, the cavities were irrigated with saline and dried with paper points. The retrograde filling materials were mixed according to the manufacturers' instructions and placed into the cavities using appropriate carriers. For MTA and One-Fil PT, an MTA carrier was used, while for Biodentine, the material was placed using the provided applicator. The filling materials were condensed with appropriate pluggers. The samples were stored in moist gauze for 24 hours at 37°C to allow setting of the materials.

## Microleakage evaluation:

All specimens were coated with three layers of nail varnish, except for the apical 1mm and allowed to dry. The specimens were then immersed in 0.5% Rhodamine B dye solution for 48 hours at 37°C. After removal from the dye, the specimens were rinsed under running tap water for 15 minutes and the nail varnish was removed using a scalpel. The roots were split longitudinally using a diamond disc under water coolant. The interface between the filling material and the canal wall was examined under a stereomicroscope (Olympus SZX16, Tokyo, Japan) at 50x magnification. Dye penetration was measured in millimeters from the apex to the maximum point of dye penetration along the filling material-tooth interface by two independent examiners who were blinded to the groups. In case of disagreement, a third examiner was consulted and the mean value was recorded.

## Statistical analysis:

Data were entered into Microsoft Excel 365 for Windows and analyzed using SPSS version 23.0 (IBM Corporation, Armonk, NY, USA). Mean, standard deviation, minimum and maximum values of microleakage were calculated for each group. The Shapiro-Wilk test was used to assess the normality of data distribution. Since the data followed a normal distribution, parametric tests were used. Two-way Analysis of Variance (2-way ANOVA) was applied to compare the microleakage values among the groups and subgroups. When 2-way ANOVA showed significant differences, the LSD post-hoc test was used for pairwise comparisons. The inter-examiner reliability was assessed using the Intraclass Correlation Coefficient (ICC). A p-value <0.05 was considered statistically significant.

## Results:

A total of 100 teeth were initially included in the study. Four teeth were excluded due to technical errors during specimen preparation, resulting in a final sample size of 96 teeth (24 per group). The Shapiro-Wilk test confirmed that the microleakage values followed a normal distribution (p>0.05). The inter-examiner reliability was excellent (ICC=0.96). The mean microleakage values, standard deviations and ranges for all groups and subgroups are presented in Table 1 (see PDF). Two-way ANOVA revealed a significant interaction effect between the filling materials and preparation techniques (F=135.345, p<0.001), indicating that the effect of the preparation technique on microleakage depended on the filling material used. Pairwise comparisons using the LSD post-hoc test showed significant differences in microleakage among the different filling materials (Table 2 - see PDF). MTA exhibited the least microleakage (0.82±0.03mm), followed by Biodentine (0.90±0.04mm) and One-Fil PT (2.29±0.19mm). The control group showed the highest microleakage (2.63±0.14mm). The differences between all materials were statistically significant (p<0.001). When comparing the preparation techniques, ultrasonic retrotip preparation showed less microleakage than conventional bur preparation across all filling materials (Table 3 - see PDF). However, this difference was not statistically significant for MTA (p=0.062) and Biodentine (p=0.058), but was significant for One-Fil PT (p<0.001) and the control group (p<0.001).

## Discussion:

The success of surgical endodontics largely depends on the quality of the apical seal achieved through retrograde filling [16]. This study evaluated and compared the microleakage of three different retrograde filling materials (MTA, Biodentine and One-Fil PT) using two different cavity preparation techniques (conventional bur and ultrasonic retrotip). The results demonstrated that MTA exhibited the least microleakage, followed by Biodentine and then One-Fil PT, regardless of the preparation technique used. Additionally, ultrasonic preparation showed less microleakage than conventional bur preparation, though this difference was only statistically significant for One-Fil PT and the control group. The superior sealing ability of MTA observed in this study is consistent with previous research [17]. MTA's excellent sealing properties can be attributed to its hydrophilic nature, which allows it to set in the presence of moisture and its ability to form hydroxyapatite crystals when in contact with tissue fluids [18]. These properties create a chemical bond between the material and dentin, reducing microleakage [19]. Additionally, MTA has a slight expansion during setting, which may contribute to better adaptation to the cavity walls [20]. Biodentine, which also showed good sealing ability in this study, is a tricalcium silicate-based material similar to MTA but with some modifications [21]. The shorter setting time and improved handling properties of Biodentine make it a practical alternative to MTA [22]. However, the slightly higher microleakage observed with Biodentine compared to MTA in this study may be attributed to differences in particle size, chemical composition and setting reaction [23, 24]. One-Fil PT, a newer tricalcium silicate-based material, showed significantly higher microleakage compared to both MTA and Biodentine in this study. This could be due to differences in composition, particle size, or setting characteristics [25]. The higher microleakage observed with One-Fil PT suggests that further improvements in its formulation may be necessary before it can be recommended as a reliable retrograde filling material [26]. These findings highlight the importance of thorough evaluation of new materials before clinical application [27]. The results of this study also demonstrated that ultrasonic retrotip preparation resulted in less microleakage than conventional bur preparation across all filling materials. This finding is consistent with previous studies that reported better adaptation of filling materials to cavities prepared with ultrasonic tips [28]. The superior performance of ultrasonic preparation can be attributed to several factors. First, ultrasonic tips produce more precise cavity preparations with parallel walls and better access, allowing for better adaptation of the filling material [29]. Second, ultrasonic preparation generates less debris and smear layer compared to conventional burs, potentially improving the bond between the filling material and dentin [30]. Third, the cavitation effect of ultrasonic irrigation may enhance the cleaning of the cavity, further improving the adaptation of the filling material [31]. The significant difference in microleakage between preparation techniques observed for One-Fil PT and the control group but not for MTA and Biodentine may be related to the physical properties of these materials. MTA and Biodentine have bioactive properties that allow them to form a chemical bond with dentin, potentially compensating for minor irregularities in cavity preparation [32]. In contrast, One-Fil PT may be more sensitive to cavity configuration, explaining why the preparation technique had a more significant impact on its sealing ability [33]. The 90-degree root resection angle used in this study was chosen based on previous research suggesting that this angle provides better stress distribution and reduces the risk of exposing dentinal tubules [34, 35]. Additionally, the 3mm resection depth was selected to remove the majority of apical ramifications and lateral canals, as suggested by anatomical studies [36]. The dye penetration method used in this study to evaluate microleakage has been widely used in endodontic research due to its simplicity and cost-effectiveness [37]. However, it has some limitations, including the subjective nature of measurement and the potential for dye diffusion not related to actual leakage pathways [38]. Despite these limitations, the method remains valuable for comparative studies of different materials and techniques [39]. The results of this study should be interpreted considering its *in vitro* nature. While efforts were made to simulate clinical conditions, factors such as masticatory forces, oral environment and biological responses could not be replicated [40].

## Conclusion:

MTA continues to provide the most reliable sealing ability among retrograde filling materials, outperforming both Biodentine and One-Fil PT. Ultrasonic preparation offers an advantage over conventional techniques, particularly for materials with weaker sealing performance. Overall, these findings reinforce MTA as the preferred retrograde material and support the use of ultrasonic preparation to enhance clinical outcomes.
